# Hello from the other side: Robust contralateral interference in tactile detection

**DOI:** 10.3758/s13414-023-02801-6

**Published:** 2023-10-23

**Authors:** Flor Kusnir, Slav Pesin, Ayelet N. Landau

**Affiliations:** Departments of Psychology and Cognitive Science, The Hebrew University of Jerusalem, Jerusalem, Israel

**Keywords:** Tactile detection, Interference, Somatosensation, Change detection, Psychophysics

## Abstract

Touch is unique among the sensory modalities in that our tactile receptors are spread across the body surface and continuously receive different inputs at the same time. These inputs vary in type, properties, relevance according to current goals, and, of course, location on the body. Sometimes, they must be integrated, and other times set apart and distinguished. Here, we investigate how simultaneous stimulation to different body sites affects tactile cognition. Specifically, we characterized the impact of irrelevant tactile sensations on tactile change detection. To this end, we embedded detection targets amidst ongoing performance, akin to the conditions encountered in everyday life, where we are constantly confronted with new events within ongoing stimuli. In the set of experiments presented here, participants detected a brief intensity change (.04 s) within an ongoing vibrotactile stimulus (1.6 s) that was always presented in a constantly attended location. The intensity change (i.e., the detection target) varied parametrically, from hardly detectable to easily detectable. In half of the trials, irrelevant ongoing stimulation was simultaneously presented to a site across the body midline, but participants were instructed to ignore it. In line with previous bimanual studies employing brief onset targets, we document robust interference on performance due to the irrelevant stimulation at each of the measured body sites (homologous and nonhomologous fingers, and the contralateral ankle). After describing this basic phenomenon, we further examine the conditions under which such interference occurs in three additional tasks. In each task, we honed in on a different aspect of the stimulation protocol (e.g., hand distance, the strength of the irrelevant stimulation, the detection target itself) in order to better understand the principles governing the observed interference effects. Our findings suggest a minimal role for exogenous attentional capture in producing the observed interference effects (Exp. 2), and a principled distribution of attentional resources or sensory integration between body sides (Exps. 3, 4). In our last study (Exp. 4), we presented bilateral tactile targets of varying intensities to both the relevant and irrelevant stimulation sites. We then characterized the degree to which the irrelevant stimulation is also processed. Our results—that participants’ perception of target intensity is always proportional to the combined bilateral signal—suggest that both body sites are equally weighed and processed despite clear instructions to attend only the target site. In light of this observation and participants’ inability to use selection processes to guide their perception, we propose that bilateral tactile inputs are automatically combined, quite possibly early in the hierarchy of somatosensory processing.

## Introduction

Our environment contains far more information than we can process. While our eyes (and ears) normally work in sync with one another, processing the same inputs simultaneously, our sense of touch does not enjoy the same harmonious coordination. Our tactile receptors are distributed across the body surface and continuously receive different inputs at the same time. Sometimes, they must be integrated (e.g., when holding a book with both hands); and sometimes they must be set apart and distinguished (e.g., holding multiple objects in both hands and suddenly feeling your phone vibrating in one of them). In this sense, the tactile modality is unique from vision and audition, which receive coherent inputs to both eyes or ears. Nonetheless, there is relatively little research about how simultaneous tactile stimulation to different parts of the body surface affect tactile cognition, particularly longer duration stimuli (>1 s).

Previous studies have shown that performance on a tactile target is hindered by the addition of other stimulation sites, particularly on the ipisilateral body side and most notably within the same hand (Evans & Craig, 1991; Evans et al., 1992; Schweizer et al., 2001; Sherrick, 1964; Tamè et al., 2011, 2014). This basic finding is at least partially explained by physiological factors (i.e., an overlapping somatosensory representation between fingers of the same hand and between body sites that are closer in somatotopic space). Tactile interference within a body side has been described and generalized over various tactile stimulation types and tasks (i.e., localization, masking, and search tasks, etc.).

The degree of perceptual interaction between contralateral body sites, particularly between fingers of opposite hands, is less understood. Several tasks have shown impairments in performance when contralateral stimulation is applied (Braun et al., 2005; D’Amour & Harris, 2014; Evans et al., 1992; Nguyen et al., 2014; Rahman & Yau, 2019; Sherrick, 1964; Tamè et al., 2011, 2014), suggesting that tactile interference effects are also observed across body sides. The attenuation of perceived intensity at the target body site has recently been proposed to be explained by divisive normalization (Rahman & Yau, 2019; see Carandini et al., 1997 and Heeger, 1992 for reviews on divisive normalization; and see Brouwer et al., 2015 for ipsilateral within-hand effects modelled by divisive normalization). Still, most bimanual tactile studies that have characterized tactile interference across body sides have not directly examined how concurrent stimulation is combined or interacts to yield the perceived intensity. In addition, other studies have shown no effects on performance or even facilitation (Craig 1985; Evans & Craig 1991; Lappin & Foulke 1973; Nguyen et al., 2014; Tamè et al., 2011) during bimanual tactile stimulation. These discrepancies may be attributed to differences in stimulation type (e.g., whether distractor and target are identical or not; see Nguyen et al. 2014; Driver and Grossenbacher 1996) as well as body site (e.g., homologous vs. nonhomologus fingers; see Halfen et al., 2020; Nguyen et al., 2014; Tamè et al., 2011), or even task-type (Tamè et al., 2014).

Importantly, most of these studies investigated the fate of tactile perception using very brief tactile targets presented within periods of no stimulation (e.g., 8–200-ms stimuli, with only one of the above studies employing a longer 800-ms stimulus; Rahman & Yau, 2019). In the present study, we embedded a brief intensity change (i.e., the target) within a relatively long vibration (1.6 ms), in order to examine individuals’ ability to detect a transient event while already engaged with an ongoing stimulus (i.e., the somatosensory system is being driven when the target is presented). Our reasons for employing a transient change within a long stimulus is two-fold. First, tactile experiences in the real world rarely result from the detection of events from a baseline of no stimulation at all, but rather from changes in the quality or intensity of an ongoing tactile perception. Second, a longer duration tactile stimulus more closely reflects the cortical dynamics present in everyday tactile performance, where neuronal responses undergo time-dependent modifications (e.g., adaptation, suppression) and engage mechanisms resulting from longer duration tactile stimulation (>1 s.; Tommerdahl, 2010). In contrast to previous studies that have extensively characterized bimanual interactions mainly involving brief tactile stimuli, we investigate their longer duration counterparts in the context of change detection.

We thus aim to extend our current understanding of bimanual tactile stimulation by investigating a new type of stimulation—a transient event embedded within a long-duration vibration. Our design presents absolute spatial certainty (i.e., the target was only delivered to the dominant index finger), and concurrent distractor stimulation to either homologous or nonhomologous contralateral body parts. To fully describe the dynamics of irrelevant tactile stimulation and how concurrent inputs are combined to yield the perceived intensity at the target body site, we manipulated: (i) the body sites to which the irrelevant stimulation was applied (homologous versus nonhomologous body parts), as well as (ii) the distance of the hands; (iii) the intensity of the irrelevant stimulation, and (iv) its content (signal-to-noise).

In our first set of experiments, we characterized and contrasted the influence of irrelevant stimulation to various body sites (contralateral index finger, pinky finger, and ankle) on ongoing perception at the dominant index finger. Thus, we were able to quantify the extent of interference between the target finger and a homologous finger, a nonhomologous finger, as well as to an entirely different body part situated in another part of anatomical space and neither close to the target hand nor within view. In Experiment 2, we examined the impact of hand distance on detection performance: Participants received irrelevant stimulation to the homologous finger, with the arm extended and occluded from view. We thus evaluated the impact of peripheral vision on the distractor body site, as well as of exogenous attentional capture. In Experiment 3, we examined how irrelevant stimulation intensity affects detection performance, in order to assess whether stimulation from the irrelevant body site generally distracts participants or whether it impacts performance in a principled way. In the last experiment (Experiment 4), we examined the impact of receiving relevant stimulation on the ignored, contralateral hand (i.e., an intensity change delivered to the irrelevant hand, concurrent with the target). The two intensity changes, though concurrent, could vary in their respective magnitudes. This design meant that the total, combined intensity-change (computed by summing the intensity changes administered to each hand) could result from various combinations of target- and distractor stimulations. Thus, we could characterize the impact of the target stimulation versus the combined stimulation on participants’ detection performance. Importantly, all experiments employed a stimulation design that included long vibrations (1.6 s) with an embedded intensity change as the target (i.e., change detection). Across this set of studies, we document robust interference between the different body sites that is invariant to finger identity, not specific to body parts, and unaffected by hand distance. Importantly, we also document an inability to ignore the irrelevant stimulation and, instead, a consistent integration between contralateral target and distractor sites that can be described as a weighted average of the concurrent stimulations, possibly indicating a pooling of sensory processes in the somatosensory system.

## Methods

The methods for the four experiments are described below. Additionally, please see Table 1 for a summary of all experimental manipulations and results.

**Table 1:**
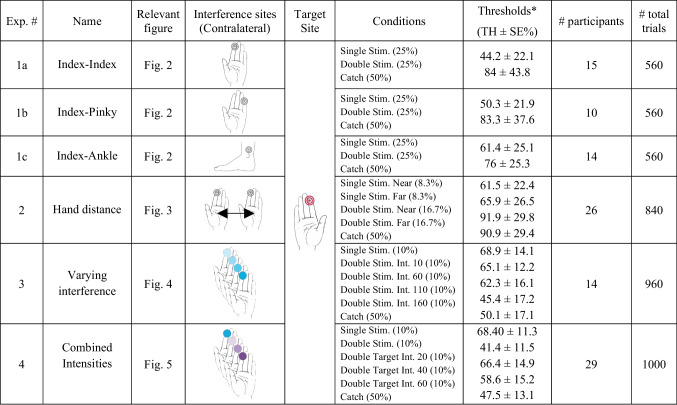
Summary of the experiments

All Experiments present average thresholds with the exception of Experiments 3-4, where we present average performance

### Experiment 1a: Homologous finger

#### Participants

We prespecified a sample size of 20 participants based on previous psychophysical and tactile interference studies. We also conducted an a priori power analysis using G*Power (Version 3.1.9.6; Faul et al., 2007), based on pilot data from our lab. The effect size (Cohen’s *d*) in our pilot data was greater than 0.8, considered to be large. With a significance criterion of α = 0.05 and a power of 0.80, the minimum sample size needed with this effect size is *N* = 15 for a design comparing differences between two dependent means (paired-samples *t* test). Twenty-one healthy participants took part in a psychophysical experiment for payment or class credit. Two participants were excluded due to a technical problem. One participant was excluded due to poor performance. Three others were excluded because the target hand turned out to be their nondominant hand. Data are thus presented for 15 participants (ages = 19–26 years, average = 23; 11 females; all right-handed). Participants signed a consent form before experimentation and the study was approved by the Institutional Review Board of Human Experimentation at The Hebrew University of Jerusalem.

#### Apparatus

Stimulation was produced with a vibrotactile coin stimulator connected to an open-source hardware, Arduino (Uno Rev3), programmed with C++ on compatible IDE. The experiment was built and run on OpenSesame (Version 3.1; Mathôt et al., 2012). The vibration produced by the Arduino was approximately 120 Hz. Headphones were used to administer white noise throughout the experiment, in order to prevent participants from hearing the vibration. Data analyses were conducted using MATLAB 2017b (The MathWorks, Inc. Natick, MA, USA) and the Palamedes Toolbox (Prins & Kingdom, 2018). This apparatus served all reported experiments.

#### Stimuli

**Fig. 1:**
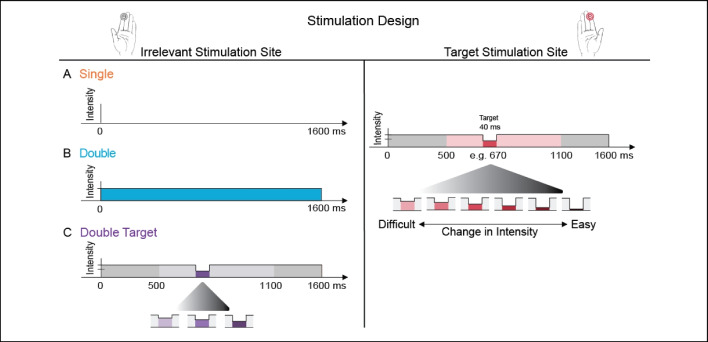
Stimulation design. In all experiments, participants received ongoing (1.6 s) vibrotactile stimulation to the index finger of their dominant hand (target stimulation site) and to an additional, contralateral body site (irrelevant stimulation site). The target, a 0.04-s change in the intensity, was embedded in half of the trials (50% catch trials). In target-present trials, detection targets appeared at a random time point between 0.5 and 1.1 s after vibration onset. The intensity change varied parametrically, resulting in seven target levels ranging from hardly detectable to easily detectable. After the end of the 1.6-s stimulation, participants were prompted to indicate via foot pedals whether or not they had detected the target. In all experiments, the target was an intensity decrement. In Experiment 4, the irrelevant stimulation site contained an intensity change that co-occurred with the target in 60% of all target-present trials. Dark red (or purple) denotes the brief decrement in stimulus intensity. Light red (or purple) denotes the time range within which targets could appear, by design. (Color figure online)

Stimulation consisted of an ongoing constant vibration that lasted 1.6 s. The detection target was embedded within the ongoing stimulation and consisted of a brief (0.04 s) decrement in intensity (Fig. 1A). We use arbitrary units (AU) to describe the intensity of the vibration and ΔAU to describe the decrement, where Δ = constant intensity − target intensity. The intensity of the ongoing constant vibration was 160 AU and that of the decrements (i.e., the targets) were parametrically varied, ranging from Δ20 to Δ140 AU, in steps of 20. This resulted in seven target intensity levels: Δ20, Δ40, Δ60, Δ80, Δ100, Δ120, Δ140, from difficult to easily detectable targets, respectively. In certain cases, two additional intensities were used (Δ10 and Δ150), but as all participants did not have these extreme values, they were excluded from analyses. Target onset was randomized from 0.5 to 1.1 s within the 1.6 s long stimulation following the onset of the vibrotactile stimulation.

#### Task

Participants were instructed to focus on a fixation cross while attending to their dominant hand (the stimulation site). In each trial, an ongoing vibrotactile stimulation was delivered to participants’ dominant index finger, at the end of which a response screen prompted them to indicate whether they had felt the embedded target or not via foot pedals. They were instructed that there would be easy and hard trials, as well as trials without a target at all (catch trials).

Participants pressed the right foot pedal for “yes” (using their right foot) and the left foot pedal for “no” (using their left foot). To keep false-alarm rates to a minimum, we instructed participants to be conservative: to only answer “yes” when they were “quite sure” that they had felt the target, and to otherwise answer “no.”

Trials in which the vibrotactile stimulation was administered only to the dominant (target) index finger are referred to as single stimulation trials. Trials in which vibrotactile stimulation was delivered to both the dominant (target) index finger, as well as to an additional (irrelevant) site (here, the homologous finger) are referred to as double stimulation, or irrelevant simultaneous stimulation (ISS) trials. The additional site, unless otherwise noted, always vibrated at the intensity of the ongoing vibration (160 AU), and for the entire duration of trial (1.6 s). It was identical to the vibration administered to the target finger, but without an embedded target (unless otherwise noted).

Single and irrelevant simultaneous stimulation (ISS) conditions were balanced (equal number of trials) but were randomized within target intensity blocks (i.e., target intensities were blocked). Each intensity block included 40 trials with a target, and 40 trials with no target (i.e., 50% catch trials per target intensity). This led to a total of 560–720 trials. The order of catch and target-present trials was randomized. The blocked target intensities were presented in randomized order, except the first, which was always the easiest (i.e., Δ140, or the second easiest, for those few participants who also performed Δ150; see Procedure).

#### Procedure

After signing an informed consent form, participants were seated in front of a computer monitor, 75 cm from the screen, with both arms positioned on the chair’s armrest, parallel to each other. Participants’ wrists were supported, but the hands themselves made no contact with the armrest (i.e., were hanging off the armrest, parallel to each other). A vibrotactile stimulator was secured to their dominant index fingertip with a customized, elastic bandage (target hand). A second vibrotactile stimulator was attached to the body site designated as the interference site (in this Experiment, the contralateral index finger) and this vibration coin vibrated with the same temporal properties as the target vibration coin, only without the embedded target. The computer monitor showed a white cross centrally positioned against a black background that served as a fixation cross throughout the experiment, and the response options appeared on either side of it, corresponding to their positions on the foot pedals (i.e., “no” on the left side of the cross and “yes” on the right).

After instructions were given, participants underwent a practice phase, consisting of 32 trials. Here, participants were familiarized with the ongoing, constant stimulation, as well as with target-present trials. Then, they completed a short practice block (32 total trials: eight trials for each of the two easiest intensities, in which half were one-hand and half were SS trials; and 16 catch trials). Practice was repeated if participants scored either <80% hit rate or >20% false-alarm rate. All participants met this criterion after a maximum of three repetitions. The experimenter remained present in the room during the practice phase.

After completing the practice, participants wore headphones and listened to white noise while they performed the experiment. All participants began with an easy target intensity (Δ140), after which performance was assessed. If they met the criterion (as in the training—i.e., ≥80% hit rate and ≤20% false-alarm rate), they continued on to the rest of the experiment. Otherwise, participants repeated this block in order to ensure that they understood the task. If they still failed to meet the criterion, they were given an even easier block (Δ150 target intensity), after which performance was re-assessed. If participants still failed to meet the criterion, they were excused from the experiment. Otherwise, they continued on to the rest of the experimental blocks, which were presented in randomized order.

The interval between trials was set to 1 s, with a 0.25 s jitter (1–1.25 s). There was a short optional break every 80 trials, which was prompted by a screen indicating that participants could take a short break and press any foot pedal to continue, when ready. At the end of the experiment, participants were either paid for their time or granted class credit.

### Experiment 1b: Nonhomologous finger

#### Participants

We prespecified a sample size of 10 participants, indicated by an a priori power analysis using G*Power (Version 3.1.9.6; Faul et al., 2007), and based on our results from Experiment 1a. The observed effect size in Experiment 1a was very large (Cohen’s *d* = −1.57), but we opted for a more conservative approach and utilized a slightly smaller effect size for our power analysis (Cohen’s *d* = 1.0). With a significance criterion of α = 0.05 and a power of 0.80, the minimum sample size needed with this effect size is *N* = 8 for a design comparing differences between two dependent means (paired-samples *t* test). Ten healthy participants took part in a psychophysical experiment for payment or class credit. Note that these were different participants as those in Experiment 1a. No participants were excluded. Data are presented for 10 participants (ages = 19–38 years, average = 24; eight females; seven right-handed). Participants signed a consent form before experimentation and the study was approved by the Institutional Review Board of Human Experimentation at The Hebrew University of Jerusalem.

#### Irrelevant stimulation site

All experimental parameters were identical to Experiment 1a, with the exception of the additional (irrelevant) stimulation site, which was the pinky finger of the nondominant hand.

### Experiment 1c: Contra-lateral ankle

#### Participants

We prespecified a sample size of 15 participants, indicated by an a priori power analysis using G*Power (Version 3.1.9.6; Faul et al., 2007), and based on our results from Experiments 1a and 1b. The observed effect sizes in both experiments was very large (Cohen’s *d* > 1), but we opted for a more conservative approach since we were moving from the opposite hand to an entirely different limb, and thus utilized a slightly smaller effect size for our power analysis (Cohen’s *d* = 1.0). With a significance criterion of α = 0.05 and a power of 0.80, the minimum sample size needed with this effect size is *N* = 10 for a design comparing differences between two dependent means (paired-samples *t* test). Fifteen healthy participants took part in a psychophysical experiment for payment or class credit. Note that these were different participants as those in the previous experiments (1a, 1b). One participant was excluded due to a technical problem. Data are presented for 14 participants (ages = 20–28 years, average = 23; 10 females; 12 right-handed). Participants signed a consent form before experimentation and the study was approved by the Institutional Review Board of Human Experimentation at The Hebrew University of Jerusalem.

#### Irrelevant stimulation site

All experimental parameters were identical to the previous experiments, with the exception of the additional (irrelevant) stimulation site, which was the ankle contralateral to the target stimulation site (i.e., the dominant hand).

### Experiment 2: Variable hand distance

#### Participants

We prespecified a sample size of 30 participants based on previous psychophysical and tactile interference studies, as well by an a priori power analysis using G*Power (Version 3.1.9.6; Faul et al., 2007), based on our results from Experiments 1a–c. The observed effect sizes in all previous experiments was very large (Cohen’s *d* > 1). However, in this experiment, we presented less repetitions per experimental condition than in the previous tasks (to accommodate the hand distance manipulation; see Table 1). Thus, we doubled the required sample size as indicated by our power analysis. With a significance criterion of α = 0.05 and a power of 0.80, the minimum sample size needed with a large effect size (Cohen’s *d* = 0.8) is *N* = 15 for a design comparing differences between two dependent means (paired-samples *t* test). Thirty-three healthy participants took part in a psychophysical experiment for payment or class credit. Note that these were different participants as those in all previous experiments. Seven participants were excluded; one due to a technical problem, two did not complete the experiment, and four due to poor performance (below chance). Data are thus presented for 26 participants (ages = 19–35 years, average = 24; 15 females; 21 right-handed). Participants signed a consent form before experimentation and the study was approved by the Institutional Review Board of Human Experimentation at The Hebrew University of Jerusalem.

#### Stimulus

Stimulus was identical to Experiment 1, with the exception that here, no extra (easier or more difficult) intensities were used, resulting in the seven target intensities specified in Experiment 1 (Δ20 to Δ140 AU, in steps of 20).

#### Task

The task was identical to Experiment 1, with the exception of (i) an additional hand distance manipulation (i.e., in which the hands were set farther apart, with the nontarget hand extended and occluded from view) and (ii) number of repetitions. With respect to (i), the experiment was divided into two main blocks: in one, the hands were positioned close together, as in Experiment 1; and in the other block, the hands were set farther apart, with the nontarget arm extended and occluded from view. The order of these two blocks (hands-close; hands-far) was counterbalanced across participants. In each block, participants were presented all seven target intensities (blocked, as in Experiment 1). Within each target intensity block, the ratio of single to double stimulation trials to catch trials was 1:2:3 (i.e., 50% catch trials, and twice the double stimulation trials as single stimulation trials). This resulted in 30 trials with no target and 30 trials with a target (20 double and 10 single stimulation trials). This led to a total of 420 trials per hand distance (hands-close, hands-far), or a total of 840 trials in the entire experiment. As in Experiment 1, the order of catch and target-present trials was randomized, as was the order of target intensities.

#### Procedure

Procedure was identical to Experiments 1, except for the practice phase, which again consisted of 32 total trials, but was split into two parts. In the first half, participants’ arms were placed as in previous experiments. In the second half, the nontarget arm was extended and occluded from view. It rested on a small platform just below shoulder height, with hands and fingers hanging off the platform. All other parameters remained the same, including the frequency of breaks.

### Experiment 3: Variable irrelevant stimulation intensity

#### Participants

We prespecified a sample size of 20 participants based on previous psychophysical and tactile interference studies, as well by an a priori power analysis using G*Power (Version 3.1.9.6; Faul et al., 2007), based on our results from Experiment 2. With a significance criterion of α = 0.05 and a power of 0.80, the minimum sample size needed with a large effect size (Cohen’s *d* = 0.8) is *N* = 15 for a design comparing differences between two dependent means (paired-samples *t* test). Since we again presented less repetitions per experimental condition (as per design), we took a conservative approach and again aimed for a slightly higher number. Twenty participants took part in a psychophysical experiment for payment or class credit. Note that these were different participants as those in all previous experiments. Six participants were excluded; one did not complete the experiment, three due to poor performance (below chance) across intensity levels; and two due to extremely high false alarms in at least one condition (69% and 100%). Data are thus presented for 14 participants (ages = 18–26 years, average = 22; 10 females; 13 right-handed). Participants signed a consent form before experimentation and the study was approved by the Institutional Review Board of Human Experimentation at The Hebrew University of Jerusalem.

#### Stimulus

Stimulus was identical to Experiments 1–2, with the exception that here, only four target intensities were used (40–100 ΔAU, in steps of 20). In addition, the intensity of the additional (irrelevant) stimulation site (i.e., nontarget hand) varied, such that there were four levels of irrelevant stimulation intensity: 160 AU, as in the previous experiments; and 110, 60, and 10 AU. Including the single stimulation condition, this resulted in five stimulation conditions: one single stimulation condition, and four double stimulation conditions.

#### Task

The task was similar to Experiments 1–2, but here the stimulation conditions were blocked (i.e., the single and the four double stimulation conditions). The single stimulation condition was always presented first, followed by the double stimulation condition with the highest stimulation intensity (i.e., 160 AU, the same double stimulation condition as was used in the previous experiments). The other three double stimulation conditions were presented in randomized order. Within each stimulation block, participants experienced the full range of target intensities (blocked and presented in randomized order). There were 20 repetitions per target intensity, and an equal number of catch trials (50% catch trials). This led to a total of 160 trials per stimulation intensity block, or 800 total trials in the experiment.

#### Procedure

Procedure was similar to the previous experiments, with the exception of the practice block. In this experiment, participants were again familiarized with the ongoing, constant stimulation, as well as with target-present trials. However, then they completed a short practice block in which four total trials were presented in the single stimulation condition only (two trials for each of the two easiest intensities, and two catch trials). Then, they completed another short practice block in which eight total trials were presented in the double stimulation condition only (four trials with the easiest two intensities and with the simultaneous [irrelevant] stimulation at the intensity of the ongoing constant vibration, i.e., 160 AU; and four catch trials). All other parameters were identical to previous experiments.

### Experiment 4: Multiple target combinations

#### Participants

We prespecified a sample size of 30 participants based on previous psychophysical and tactile interference studies, as well by an a priori power analysis using G*Power (Version 3.1.9.6; Faul et al., 2007), based on our previous experiments. With a significance criterion of α = 0.05 and a power of 0.80, the minimum sample size needed with a large effect size (Cohen’s *d* = 0.8) is *N* = 24 for a design comparing the main effects and interactions of several groups (ANOVA design). Since the comparisons of interest in this experiment were new, we took a conservative approach and again aimed for a slightly higher number. Thirty-three participants took part in a psychophysical experiment for payment or class credit. Note that these were different participants as those in all previous experiments. Three participants were excluded due to technical problems and one due to floor performance. Data are thus presented for 29 participants (ages = 19–26 years, average = 23; 11 females; 10 right-handed). Participants signed a consent form before experimentation and the study was approved by the Institutional Review Board of Human Experimentation at The Hebrew University of Jerusalem.

#### Stimulus

Stimulus was identical to the previous experiments, with the exception that here, only five target intensities were used (40–120 ΔAU, in steps of 20). Similarly to Experiments 4a and 4b, a third experimental condition was added, in which an intensity decrement was embedded in the simultaneous (irrelevant) stimulation (i.e., the nontarget finger). In this experimental condition, the intensity decrement of the non-target finger could vary in intensity (20, 40, or 60 ΔAU), but its temporal characteristics were identical to the target presented to the dominant hand (i.e., it co-occurred with the target). This experimental condition (double target) occurred in addition to the single and double stimulation conditions (see Fig. 1).

#### Task

The task was similar to previous experiments. Single, double stimulation, and double target conditions were presented in a ratio of 1:1:3 and randomized within target intensity blocks. Each intensity block included 50 trials with a target (10 repetitions each in the single and double stimulation conditions, and 10 repetitions of each possible double target intensity, leading to a total of 30 double target trials). The remaining 50 trials per intensity block contained no target (i.e., 50% catch trials). Differently from previous experiments, each intensity block was presented twice (all possible intensities were presented in randomized order, and then randomized a second time). This led to a total of 1,000 trials.

#### Procedure

Procedure was identical to previous experiments. The practice block was also identical to that in the previous experiments.

### General

#### Analyses

For each participant included in the analysis, we estimated the percentage of hits, misses, correct rejections, and false alarms for each condition (e.g., single and double stimulation conditions) separately.

For all main analyses, we used a two-tailed percentile bootstrap procedure for dependent groups, with 10,000 samples with replacement (Efron & Tibshirani, 1993; Wilcox, 2012). We compared mean detection rates between stimulation conditions (e.g., single vs. double stimulation; target intensities, collapsed) by calculating percentile confidence intervals around the mean difference between the two stimulation conditions. First, we sampled participants with replacement, keeping their corresponding mean detection rates (i.e., averaged across target intensity levels) in the two conditions. We then calculated the mean difference between conditions across all (sampled) participants. We performed these two steps 10,000 times, and each time saved the mean difference between conditions. Then, we sorted the bootstrapped means, and used the 2.5 and 97.5 percentiles to form the boundaries of the 95% bootstrap confidence intervals (for an alpha, α = 0.05). To calculate whether the detection rates in the two conditions of interest differed from each other, we estimated the overlap of the bootstrapped distribution with zero (i.e., the null hypothesis, that there is no difference in detection rates between the two stimulation conditions), in the following manner: *p* value = [one minus the percentage of bootstrap values above (or below) zero, multiplied by two (for a two-tailed test)].

To estimate psychometric functions, the responses (hit rates, or percent detection) for each participant were modelled by fitting logistic functions for each experiment, using a maximum-likelihood procedure for each condition (e.g., single, double stimulation) and experiment (Palamedes Toolbox; Prins & Kingdom, 2018). We then calculated the 50% threshold of performance, as well as the slope of the psychometric curve, for each condition separately. Both threshold (α) and slope (β) parameters were allowed to vary freely, while guess (γ = 0) and lapse (λ = 0.01) rates were fixed for all participants.

A post hoc analysis between Experiments 1a, b, and c contrasted the magnitude of interference between pairs of Experiments, in order to determine whether the impact of the irrelevant stimulation varied as a function of stimulation site (i.e., homologous index finger, nonhomologous pinky finger, and contralateral ankle). The magnitude of interference in each experiment is given by the difference in 50% thresholds between the single and double stimulation conditions in that experiment. We then used a two-tailed percentile bootstrap procedure for independent groups to contrast the difference in magnitude between all possible pairs (three). First, we sampled participants with replacement, using the minimum number of participants across the three experiments (*n* = 10, Experiment 1b). We then calculated the mean difference in 50% thresholds across all sampled participants (for each Experiment separately), and computed the difference between experiments. We performed these steps 10,000 times, and each time saved the difference between experiments. Then, we sorted the bootstrapped differences, and used the 2.5 and 97.5 percentiles to form the boundaries of the 95% bootstrap confidence intervals (as outlined earlier in this section). To calculate whether the interference magnitudes in the two experiments of interest differed from each other, we estimated the overlap of the bootstrapped distribution with zero (i.e., the null hypothesis, that there is no difference in magnitude of interference between the two experiments), in the following manner: *p* value = [one minus the percentage of bootstrap values above (or below) zero, multiplied by two (for a two-tailed test)].

In Experiment 3 (varying ISS intensity), we modelled the relationship between the ISS intensity and target-detection performance using a simple linear regression. This analysis was performed for each target intensity separately. Thus, for a given target intensity we fitted a first-degree polynomial to detection performance over the different ISS intensity levels (see Fig. 4B). This was performed for each participant separately and yielded four slopes per participant (one for each target intensity). Then, we resampled participants with replacement, and derived a sampling distribution of average-slope values for each target intensity (with 2.5 and 97.5 percentiles to form the boundaries of the 95% bootstrap confidence intervals).

To calculate whether these slopes differed from those that would be observed if ISS intensity did not significantly impact target-detection performance, we computed a bootstrap sampling distribution based on shuffling ISS intensity labels and generating slopes based on the shuffled data. This surrogate analysis was also performed within each target intensity level separately.

First, we shuffled ISS intensity labels for each participant, and computed the slope using the linear regression method described above. We repeated this procedure 10,000 times and averaged the slopes across participants for each iteration. This results in a distribution of group-averaged slopes. This bootstrap sampling distribution was contrasted with the distribution of the original slope data, by computing the overlap.

In Experiment 4, we fitted a Bayesian binomial logistic regression model in order to examine the influence of the combined intensity decrement on detection rates (implemented by R’s package brms using Stan; Burkner, 2018; Stan Development Team, 2020). First, the combined intensity decrement was calculated by summing the target intensity delivered to the target hand and the co-occurring intensity decrement embedded in the irrelevant stimulation (i.e., in the double target condition); or 0, in the case of the double stimulation condition (where there was no intensity decrement embedded in the ongoing irrelevant stimulation; see Fig. 5, bottom panel). The dependent variable was detection (i.e., trials in which participants successfully detected the target) and the model included fixed effects of stimulation condition (five total: single stimulation, double stimulation, and the three double target conditions), combined intensity decrement (summed across both hands, as described above), and their interaction. The random-effect structure included a by-subject random intercept and random slopes for the same predictors (stimulation condition, combined intensity decrement, and their interaction). We report the mean estimates, standard errors and the 95% credible intervals for all relevant model parameters. In addition, for the experimental questions of interest, we report Bayes factors using the hypothesis function of the brms package, which calculates the Savage–Dickey density ratio between the prior and the posterior for each hypothesis. We used the default priors set by the brms package for all coefficients and group-level random effects (weakly informative priors).

## Results

### Experiment 1a: Homologous finger

**Fig. 2:**
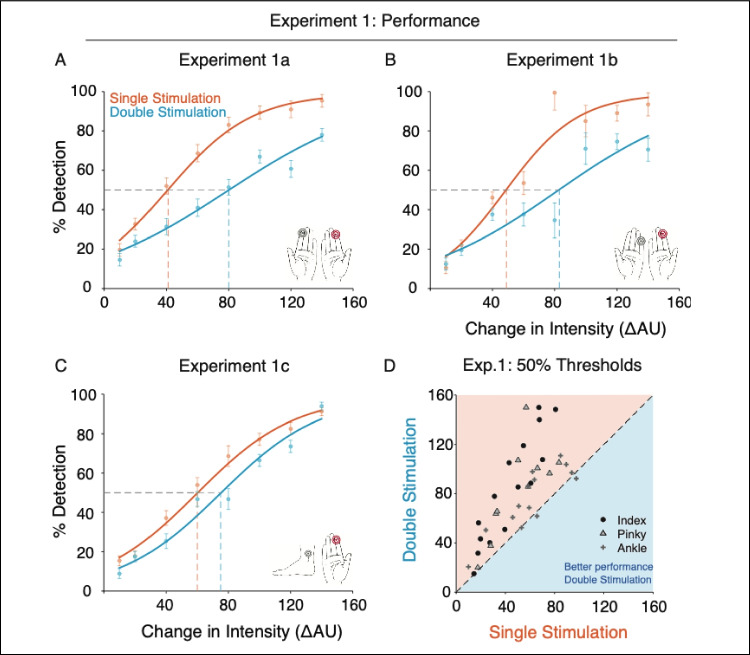
Detection performance for Experiment 1. Mean psychometric functions in the single and double stimulation conditions for Experiments 1a (**A**), 1b (**B**), and 1c (**C**). In all three experiments, participants’ ability to detect tactile targets while receiving additional (irrelevant) stimulation to a contralateral body site was significantly hindered. The scatterplot (**D**) shows the 50% thresholds for individual participants (single points) in all three experiments. Nearly all participants exhibited lower thresholds in the single stimulation condition (almost all participants to the left of the identity line). (Color figure online)

Figure 2A (top left panel) shows the mean psychometric functions in the single and double (homologous index finger) stimulation conditions. As per experimental design, participants’ performance increased as a function of increasing target intensities, from nearly no target detection at the hardest intensity levels to almost perfect target detection at the easiest intensity levels. To examine the effects of irrelevant stimulation on target detection, we first compared overall mean detection rates between the two stimulation conditions (single, double); and subsequently the 50% thresholds of each.

For all main analyses, we used a two-tailed percentile bootstrap for dependent groups, with 10,000 samples with replacement. We compared overall mean detection rates between the two stimulation-conditions (single vs. double stimulation; target intensity, collapsed) by calculating percentile confidence intervals around their difference (see Methods; General; Analyses). Square brackets indicate the boundaries of the 95% confidence intervals (CI) constructed from this analysis.

In Experiment 1a, participants generally exhibited higher detection rates when targets were presented to the dominant index finger with no additional simultaneous stimulation, compared with when they received irrelevant simultaneous stimulation to the homologous finger (single minus double stimulation = 22.7% [16.7, 28.6], *p* < .001).

In addition, we compared target intensities corresponding to the 50% detection rate in each stimulation condition, as estimated from individual participants’ psychometric fits. Here too, participants exhibited lower detection thresholds in the single stimulation condition (mean 50% threshold, single minus double stimulation = −39.8 ΔAU [−52.0 −27.6], *p* < .001). This difference corresponds to that of two target-intensity levels higher in the double stimulation condition. All participants showed this pattern (see Fig. 2D).

In addition, the difference in performance between stimulation-conditions also manifested as a difference in slopes between individual participants’ psychometric fits, such that the slope of the single stimulation condition was consistently higher than that of the Double stimulation condition (mean slopes, single minus double stimulation: 0.027 [.008 .051], *p* < .005). There was no difference in false alarms between stimulation conditions (mean false-alarm rate across the entire experiment, 13.8 % ± 9.8, *p* = 0.4).

### Experiment 1b: Nonhomologous finger

In Experiment 1b, we conducted the same experiment as above, but placed the additional vibrotactile stimulator on a nonhomologous finger: the contralateral pinky finger. Not only is this the farthest possible finger from the dominant index finger, but it is also served by a different dermatome. In order to rule out interference effects resulting from sensory overlap in the spinal tract (e.g., due to decussation), we opted to stimulate a digit connected to an entirely different spinal nerve.

Figure 2B (top right panel) shows the mean psychometric functions in the single and double (contralateral pinky finger) stimulation conditions. As per experimental design, participants’ performance again improved as a function of increasing target intensities. Like in Experiment 1a, we compared overall mean detection rates between the two stimulation conditions, and subsequently the 50% thresholds of each, in order to examine the effects of the irrelevant stimulation on target detection.

Participants again exhibited higher detection rates when targets were presented to the dominant index finger without additional stimulation, compared with when they received irrelevant simultaneous stimulation to the contralateral pinky finger (single minus double stimulation = 19.9% [14.5, 26.2], *p* < .001).

Participants also exhibited lower detection thresholds in the single compared with the double stimulation condition (mean 50% threshold, single minus double stimulation = −33.0 ΔAU [−49.9, −19.8], *p* < .001). This difference corresponds to that of one-to-two target-intensity levels higher in the double stimulation condition. All participants showed this pattern (see Fig. 2D).

The difference in performance between stimulation-conditions also manifested as a difference in slopes between individual participants' psychometric fits, such that the slope of the Single Stimulation condition was consistently higher than that of the Double Stimulation condition (mean slopes, Single minus Double Stimulation: 0.038 [.023, .056], p < .001). There was no difference in false alarms between stimulation-conditions (mean false alarm rate across the entire experiment, 8.3 % ± 6.1, p = 0.6).

### Experiment 1c: Contralateral ankle

Experiment 1c was identical to the previous experiments, but here the irrelevant simultaneous stimulation was administered to the contralateral ankle.

Figure 2C (bottom left panel) shows the mean psychometric functions in the single and double (contralateral ankle) stimulation conditions. As per experimental design, participants’ performance again improved as a function of increasing target intensities.

Like in the previous experiments, participants exhibited higher detection rates when targets were presented to the dominant index finger without additional stimulation, compared with when they received simultaneous stimulation to the contralateral ankle (single minus double stimulation = 7.9% [4.2, 11.6], *p* < .001). This difference in detection performance also manifested as lower detection thresholds for the single stimulation condition (mean 50% threshold, single minus double stimulation = −14.6 ΔAU [−21.2 −8.1], *p* < .001). This difference corresponds to that of almost one target-intensity level higher in the Double stimulation-condition. Nearly all participants showed this pattern (see Fig. 2D).

In contrast to the previous experiments, there was no difference in slopes or false alarms between stimulation conditions (mean slope across the entire experiment, 0.04 ± 0.02, *p* = 0.4; mean false-alarm rate across the entire experiment, 8.2 % ± 6.5; *p* = 0.2).

### Experiment 2: Variable hand distance

In this experiment, participants underwent the same protocol as in Experiment 1a (i.e., the irrelevant stimulation site was the homologous index finger), but during half of the experiment, the irrelevant stimulation site (i.e., the nontarget arm) was extended and occluded from view.

**Fig. 3:**
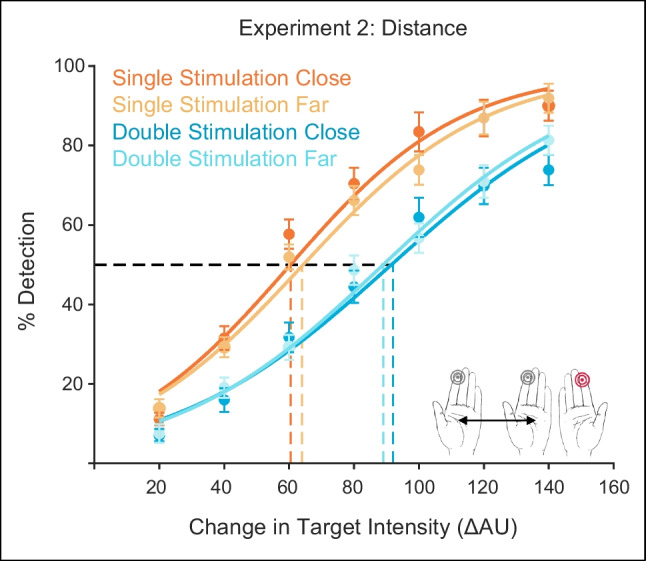
Detection performance for Experiment 2. Mean psychometric functions in the Single and Double Stimulation conditions for Experiment 2, in Near and Far arm distances (dark orange and dark blue, or light orange and light blue respectively). In both hand distance conditions, participants’ ability to detect tactile targets while receiving additional stimulation to the homologous finger was significantly and equally hindered. (Color figure online)

Figure 3 shows the mean psychometric functions in both single and double stimulation conditions, in near and far arm distances (i.e., both hands in front of the body, as in Experiment 1; or nontarget arm extended and behind the body, occluded from view). As per experimental design, participants’ performance increased as a function of increasing target intensities, from nearly no target detection at the hardest intensity levels to almost perfect target detection at the easiest intensity levels.

We assessed whether the position of the hands (near, far) impacts the magnitude of detection interference. To this end, we first compared overall mean detection rates (target intensity, collapsed) between the two stimulation conditions when the arms were positioned far apart (single vs. double stimulation, hands-far). We observed a robust impact on target detection, as observed in all previous experiments (single minus double stimulation, hands-far = 14.3 % [11.3, 17.9], *p* < 0.001). In addition, and as observed in Experiment 1, we observed a robust impact on target detection when the hands were positioned close together (single minus double stimulation, hands-near, 18.0 % [15.2, 20.9], *p* < 0.001).

Within stimulation conditions, the overall mean detection rates between far and near hand distances did not differ (double stimulation: hands-far minus hands-near, *p* = 0.6; single stimulation: hands-far minus hands-near, *p* = 0.3).

We then compared target intensities corresponding to the 50% detection rate, as estimated from individual participants’ psychometric fits. Participants exhibited lower detection thresholds in the single compared with the double stimulation condition, in both hand distances (50% thresholds; single minus double stimulation, hands-far: −25.1 ΔAU [−31.5 −19.2], *p* < .001; hands-near: −30.4 ΔAU [−35.7 −25.2], *p* < .001). As observed in Experiment 1, this difference in 50% thresholds corresponds to that of one-to-two target-intensity levels higher in the double stimulation condition. Within stimulation conditions, the 50% thresholds did not differ between the two hand distance conditions (double stimulation, *p* = 0.8; single stimulation, *p* = 0.2).

The above differences in detection performance also manifested as a difference in slopes between individual participants’ psychometric fits, such that the slope of the single stimulation condition was consistently higher than that of the double stimulation condition (mean slopes; single minus double stimulation, hands-far: 0.013 [.0043 .0234], *p* = .003; hands-near: 0.018 [0.0072, 0.0297], *p* = .001). Within stimulation conditions, the slopes did not differ between hand distance (double stimulation, *p* = 0.6; single stimulation, *p* = 0.4).

Unlike in previous experiments, participants exhibited different false alarm rates between stimulation conditions, such that there were more false alarms in the single compared with the double stimulation condition (mean false-alarm rate across the entire experiment, 8.23 % ± 7.9; single minus double stimulation, hands-far: 3.02 % [1.51, 4.67], *p* < .001; hands-near: 4.26 % [2.55, 6.21], *p* < 0.001). Within stimulation-conditions, there was no difference in false alarms between hand distance (double stimulation, *p* = 0.7; single stimulation, *p* = 0.2).

#### Interim summary

In Experiments 1–2, we examined the impact of irrelevant tactile stimulation on tactile detection in a constantly attended location. We found a compelling effect of interfering stimulation on detection performance for all body sites that we measured (homologous and nonhomologous fingers, and contralateral ankle).

In order to assess whether the effects of detection interference differed as a function of stimulation site, we contrasted the magnitude of interference between Experiments 1a, b, and c. The magnitude of interference is given by the difference in 50% thresholds between stimulation conditions. This analysis indicated that the impact of interference between Experiments 1a (homologous finger) and 1b (nonhomologous finger) did not differ (mean 50% thresholds; Experiment 1b minus Experiment 1a = 6.78 ΔAU [−14.5, 27.3], *p* = 0.5). However, the magnitude of interference when the additional stimulation was applied to the contralateral ankle (Experiment 1c) was significantly less than that of Experiments 1a and 1b (mean 50% thresholds; Experiment 1c minus Experiment 1a = 25.2 ΔAU [8.2, 42.6], *p* < .003; Experiment 1c minus Experiment 1b = 18.4 ΔAU [8.3, 42.3], *p* = .003).

In summary, the impact of irrelevant tactile stimulation on detection performance was not modulated by finger identity or finger distance from the target site, but it was alleviated by applying the additional stimulation to the contralateral ankle—in the order of one target intensity level lower. In addition, the impact of irrelevant tactile stimulation on detection performance was not modulated by hand distance: even when the nontarget hand was fully extended and occluded from view, detection performance did not change as compared with when the hand was in front of the body and within view. In the following two experiments, we first further characterize the parametric nature of the pooling of inputs from the attended and the unattended body side by varying the irrelevant stimulation intensity in Experiment 3. We then directly address the question of how relevant and irrelevant inputs are weighted by embedding not only ongoing stimulation (i.e., noise) in the irrelevant site, but also intensity decrements which coincided with the target (i.e., signal) in the attended hand.

### Experiment 3: Variable irrelevant stimulation intensity

In this experiment, participants underwent a similar protocol as in Experiment 1a (i.e., the irrelevant stimulation site was the homologous index finger). However, participants performed the experiment with only four target intensities, and with an additional experimental manipulation in which the irrelevant simultaneous stimulation (ISS; i.e., to the nontarget hand) varied in intensity. Thus, the non-target hand could vibrate at one of four different intensity levels (10, 60, 110, or 160 AU). We examined the impact of the double stimulation intensity on detection performance.

**Fig. 4:**
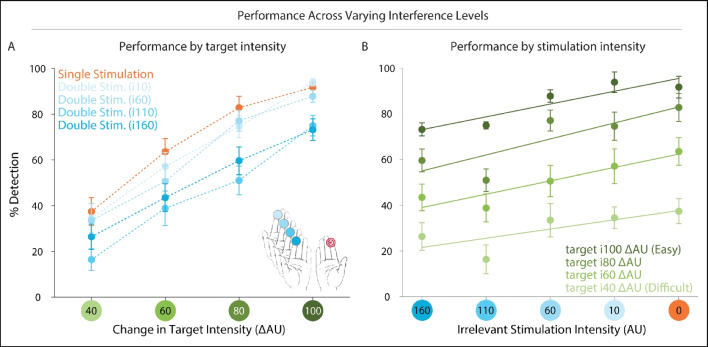
Detection performance in Experiment 3. Participants’ ability to detect tactile targets varied as a function of target intensity (**A**) and irrelevant stimulation intensity (**B**). Participants received different levels of irrelevant stimulation intensity to the homologous index finger. For each irrelevant stimulation intensity, participants exhibited higher detection rates as target intensity increased (**A**). The relationship between detection-performance and irrelevant stimulation intensity can be modelled as a simple linear regression, for each target intensity level (**B**), such that lower irrelevant stimulation led to increased detection-performance, except for in the weakest target intensity level (**B**, lightest green line). (Color figure online)

Figure 4A shows the mean performance across target intensity levels for the five stimulation conditions (single stimulation; and the four double stimulation intensity levels described above). As per experimental design, participants’ performance increased as a function of increasing target intensities, from very little or below-chance target detection at the hardest intensity levels to greater target detection at the easiest intensity levels.

In order to estimate the impact of irrelevant stimulation intensity on detection performance, we modelled the relationship between the two using a simple linear regression (see Fig. 4B). This analysis was performed for each target intensity level, separately. A comparison between the derived slopes and a bootstrap null distribution of slopes (see Methods; General; Analyses) indicated that detection-performance increased linearly with decreased irrelevant stimulation intensity, except for in the weakest target intensity (see Fig. 4B, lightest green line) (mean slopes across participants: 100 ΔAU = 0.14 [.11, .21], *p* < .001; 80 ΔAU = 0.16 [.11, .21], *p* < .001; 60 ΔAU = 0.13 [.063, .20], *p* < .003; 40 ΔAU, *p* = 0.6).

### Experiment 4: Multiple target combinations

In this experiment, participants underwent a similar protocol as in Experiment 1a (i.e., the irrelevant stimulation site was the homologous index finger). However, we added a third stimulation condition, referred to as the double target condition. In this condition, an intensity decrement of varying magnitudes was embedded in the irrelevant stimulation and co-occurred with the target (see Fig. 1C).

**Fig. 5:**
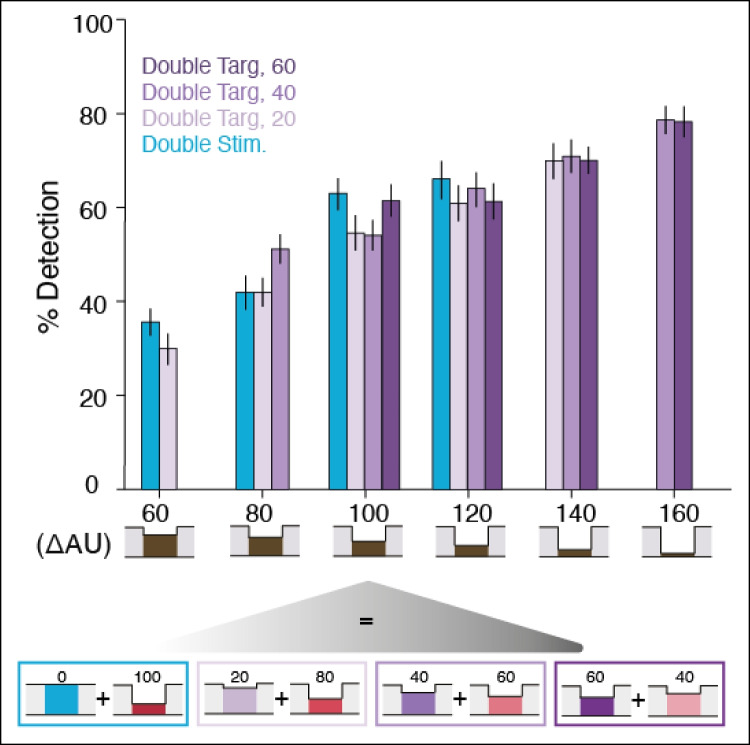
Detection performance according to summed hands. Mean detection performance in the double stimulation and double target conditions for Experiment 4. The target intensity of the target hand (depicted in varying shades of red) and the intensity decrement that co-occurred with the target but was embedded in the irrelevant stimulation (depicted in blue and purple) were summed. This resulted in six possible combined intensities (*x*-axis). Despite explicit instructions to perform only on the target hand and to ignore the irrelevant stimulation on the other hand, participants consistently exhibited similar detection rates within any given combined intensity level. This suggests that they were unable to ignore the distractor site and performed according to the input to both stimulation sites. (Color figure online)

Our aim was to characterize the impact of the combined inputs to the attended and irrelevant stimulation sites on detection performance. To this end, we grouped together all stimulation conditions that resulted in a single combined decrement when the target and irrelevant hands were summed (see Fig. 5). We then assessed the impact of the combined decrement, as well as of stimulation condition, on detection performance using a Bayesian binomial logistic regression. We report the mean estimates, standard errors and the 95% credible intervals for all relevant model parameters. In addition, for the experimental questions of interest, we report Bayes factors to quantify the extent to which the data supports our hypotheses.

In comparison to the double stimulation condition (dummy coded as the “baseline” condition, i.e., to which all other conditions are compared), and as expected based on the previous reported experiments, the single stimulation condition resulted in better detection performance (*ME* = 0.44, *SE* = 0.14, Bayesian 95% CI [0.16, 0.70]). More importantly for our experimental question, the combined decrement significantly predicted target detection in the double stimulation condition (*ME* = 0.36, *SE* = 0.04, Bayesian 95% CI [0.29, 0.43]). Only in the single stimulation condition did it have a stronger effect on performance, as measured by the interaction (*ME* = 0.20, *SE* = 0.04, Bayesian 95% CI [0.12, 0.29]). We then computed evidence ratios for the hypothesis that the combined decrement does not affect detection rates differently in each of the three double target conditions (Fig. 5, shades of purple, 60, 40, and 20) as compared with the double stimulation (Fig. 5, blue bars) (1/evidence ratio = 0.0067, 0.0048, and 0.0034 respectively; all very strong evidence that combined-decrement levels affected all of the double stimulation conditions similarly). In addition, detection rates generally increased with increasing combined-decrement levels (1/evidence ratio = −3.09e23; very strong evidence in favour of an impact of combined-decrement level on detection rates in all double stimulation and double target conditions). Last, detection rates were affected more strongly by the combined-decrement level in the single stimulation condition compared with all other conditions (1/evidence ratio = 8.07e14, very strong evidence that the single stimulation condition is not equivalent to the rest).

## Discussion

In this set of studies, we investigated how simultaneous stimulation to two sites on opposite sides of the body impact tactile detection. To this end, we ran a series of experiments in which individuals had to detect an intensity decrement within an ongoing vibration on their dominant index finger, while receiving irrelevant stimulation to another body site. We asked whether concurrent tactile inputs—one always relevant and the other always irrelevant—can be differentiated in accordance with ongoing goals.

We found that participants’ ability to detect tactile targets was impaired regardless of whether distractor vibration was applied to the homologous or nonhomologous effector, or even to a different limb. In Experiment 1(a–c), all participants clearly displayed reduced detection rates and higher detection thresholds compared with single-site stimulation, meaning that the irrelevant stimulation interfered with their ability to detect the target (see Fig. 2). As shown in previous studies involving a wide variety of tasks (e.g., discrimination, localization, go/no-go protocols), our participants exhibited a complete inability to ignore the irrelevant stimulation. Interestingly, however, the magnitude of this interference was invariant to finger identity (i.e., to whether the irrelevant stimulation was applied to the homologous or nonhomologous finger of the opposite hand). The observed interference was significantly greater in magnitude when produced by the contralateral hand (homologous or nonhomologous finger) compared with the contralateral ankle, which is more distant both in physical space and in the neural representation. This finding is surprising given previous studies showing tactile interactions as a function of somatotopic distance between the stimulated fingers of opposite hands (Braun et al., 2005; Tame et al., 2011). However, it could perhaps be understood by considering two features of cortical representation that directly affect the integration and differentiation of tactile inputs: the topographic selectivity and laterality of the somatosensory cortex (Saadon-Grosman et al., 2020). *Topographic selectivity* indicates how specific a cortical response is to a particular body part, and *laterality* reflects the extent to which the two sides of the body are processed independently. Recent imaging efforts of the somatosensory homunculus reveal several topographic maps which diverge on these parameters (Saadon-Grosman et al., 2020), containing representations for the ipsilateral body side and for different parts of the body surface. For example, while BA 3b (an area within S1) is characterized by specialized finger response, subsequent S1 processing areas (e.g., BA 1 and BA 2) are less selective. Here, receptive fields are less finely tuned, and cortical responses are elicited by neighbouring digits, not just by a preferred digit (Iwamura, 1998; Martuzzi et al., 2014; Saadon-Grosman et al., 2020). Diminished laterality in the somatosensory system is also supported by a wide variety of cognitive studies, ranging from neural adaptation between homologous fingers (Tamè et al., 2012) to interference between homologous fingers in detection tasks (Tamè et al., 2011). Similarly, tactile training has been shown to transfer from a trained finger to either neighbouring or homologous fingers of the opposite hand (Harrar et al., 2014; Harris et al., 2001). Our psychophysical investigation may be a manifestation of the functional consequences of the architecture of such somatosensory maps.

In addition, we describe detection rates that can be predicted by the equally weighted summation of inputs to both relevant and irrelevant stimulation sites. When receiving bimanual stimulation, participants are unable to ignore the distractor site, as instructed; instead, they perform according to the combined inputs to both hands. This results in similar detection performance between targets that, for example, are normally difficult to detect (but are accompanied by a larger intensity decrement in the irrelevant stimulation) and targets that are well above threshold (but are accompanied by a small intensity decrement in the irrelevant stimulation). The seemingly obligatory and equivalent pooling of tactile inputs occurs despite absolute spatial certainty over the target site and attentional allocation to it. Importantly, however, bimanual stimulation always resulted in interference (in detection rates) as compared with the single stimulation condition. This finding suggests that multiple inputs are always likely characterized by higher levels of noise (or lower signal-to-noise).

### Further characterizing tactile interference

Experiment 2 (variable hand distance) sought to address the impact of hand distance and the proximity of the hands in space on the observed interference effects of Experiment 1. One possible explanation for the decrement in tactile detection is that the irrelevant stimulated body site simply cannot be ignored, thus eliciting sensory competition between the two body sites (Johansen-Berg & Lloyd, 2000). Thus, in Experiment 2, participants’ nontarget arm was extended and occluded from view, minimizing the potential role of proprioceptive input and of involuntary attentional capture. Attentional shifts between body parts have been shown to take time in tactile detection tasks (Lakatos & Shepard, 1997). In addition, numerous studies have reported that noninformative vision of a body part (i.e., in this experiment, the target arm) improves tactile detection thresholds and discrimination performed on that body part, potentially by affecting tactile receptive fields (Haggard et al., 2007; Kennett et al., 2001; Schaefer et al., 2005). Thus, we might expect the occlusion of the irrelevant arm to result in attentional facilitation or in an increased sensitivity in the target hand, leading to enhanced performance when the hands were further apart. However, in our experiment individuals’ tactile performance was unaffected by the distance between hands. Even with the contralateral arm extended and occluded from view (i.e., was further away and in a different part of space than the target hand), the detrimental effects of irrelevant stimulation remained unchanged. The observed tactile interference is unaffected by the proximity of the hands in space.

### Varying signal and noise in the irrelevant stimulation

Experiments 3 and 4 allowed us to understand detection performance under different combinations of signal (target hand) and noise (irrelevant hand). In Experiment 3, we parametrically varied the intensity of the irrelevant stimulation, such that in some experimental blocks it was less intense (i.e., lighter) and in others more intense (i.e., closer to the ongoing intensity of the target hand). As the intensity of the irrelevant stimulation increased, individuals exhibited worse detection performance (see Fig. 4). In fact, higher levels of noise masked the target-signal in a linearly increasing manner. It has previously been shown in experiments employing brief tactile events that participants are generally unable to restrict attention to only the target site (Craig, 1972; Green & Craig, 1974). Here, we replicate these findings and extend them by applying distractor stimulation to the contralateral body site and by characterizing its impact on target detection as a linearly increasing interference.

Experiment 4 complemented these results by giving us insight into how concurrent tactile stimulation interacts when the irrelevant body site contains target-like information (i.e., a concurrent intensity change of varying magnitudes). In this experiment, target detection in the context of irrelevant stimulation (i.e., all conditions except the single stimulation) reflected a simple summation between stimulation from the two index fingers, invariant to the specific combination of intensity decrements embedded in each. In other words, a combined intensity decrement of 80 ΔAU, whether resulting from 20 ΔAU to the irrelevant finger and 60 ΔAU to the relevant one, or 40 ΔAU to each, led to equivalent detection rates (see Fig. 5). This indicates, not only that participants were wholly unable to ignore the irrelevant stimulation, but also that they consistently perform according to the summed signal of both hands, and with equivalent weighting. This finding suggests pooling of or integration between concurrent stimuli of opposite hands, despite explicit instructions to attend and to perform solely on one hand. In order to fully validate this possible account of bimanual interactions, it would first be important to generalize this finding in additional experimentation, by examining other limb configurations like hand alignment and body posture. Other possible accounts of tactile interactions during simultaneous concurrent stimulation have been proposed, most notably divisive normalization (Rahman & Yau, 2019; see Brouwer et al., 2015; Carandini et al., 1997; Heeger, 1992). Summation and normalization model comparison could help elucidate which best accounts for contralateral bimanual interactions, for both brief and long-duration tactile events.

## Conclusion

Understanding how multiple tactile inputs interact in the context of change detection informs perception under more natural conditions: In our daily life, the tactile receptors across our skin surface, from our feet to our hands, are constantly stimulated by different objects, and depending on our current goals, they must sometimes be coordinated, and sometimes set apart. Most previous tactile studies examining multisite stimulation have examined short-duration targets (in the order of .01–.2 s) presented in silence or multiple targets presented within rapid succession. In our study, we examined individuals’ ability to detect a brief change within a relatively longer (>1 s) ongoing vibrotactile stimulus in a known and constantly attended location. Our choice of task enabled a direct examination of how well a tactile target is detected in light of irrelevant stimulation. Participants were explicitly instructed to ignore the irrelevant stimulation site and to detect the target, thus enabling an examination of contralateral stimulation with complete certainty over target location, and with ongoing bilateral engagement of the somatosensory system.

Across the set of experiments reported here, we characterize compelling and robust tactile interference when receiving irrelevant stimulation to the opposite body side during detection performance on a known, attended body part. This interference is consistently present across individuals and is not modulated by changes in hand distance or by the specific combination of target intensities presented to each body site, pointing to obligatory interactions and possibly early pooling of tactile inputs that results in an integrated percept.

Recent notions on the organization of somatosensory maps suggest that, contrary to former belief, somatosensory representations are not as specific or as lateralized as previously thought (Saadon-Grosman et al., 2020). Our work supplements this neural response characterization with a thorough description of its functional consequences to detection in somatosensation. Consistent with reduced laterality, we suggest a robust and early locus of sensory integration from across body sides in the tactile domain. The rules for integration across body sides are possibly described by a simple summation, rather than by more complex interactions between fingers. Future studies combining time resolved neuroimaging with sophisticated psychophysics might shed light on the precise locus and rules of integration in the somatosensory system.

## Data Availability

The data or materials for the experiments reported here are available upon request, and none of the experiments was preregistered*.*
